# Health care leaders’ perspectives on the business impact of mobile health clinics

**DOI:** 10.1186/s12939-023-01982-8

**Published:** 2023-09-01

**Authors:** Mollie M. Williams, Sarah T. Bui, Josephina S. Lin, Gregory H. Fan, Nancy E. Oriol

**Affiliations:** Harvard Medical School, 200 Longwood Ave, Boston, MA 02115 USA

**Keywords:** Mobile clinics, Health care finance, Health care delivery

## Abstract

**Background:**

By analyzing how health care leaders in the United States view mobile health programs and their impact on the organization’s bottom line, this study equips those who currently operate or plan to deploy mobile clinics with a business case framework. Our aim is to understand health care leaders’ perspectives about business-related incentives and disincentives for mobile healthcare.

**Methods:**

We conducted 25 semi-structured key informant interviews with U.S. health care leaders to explore their views and experiences related to mobile health care. We used deductive and inductive thematic analysis to identify patterns in the data. An advisory group with expertise in mobile health, health management, and health care finance informed data collection and analysis.

**Results:**

In addition to improving health outcomes, mobile clinics can bolster business objectives of health care organizations including those related to budget, business strategy, organizational culture, and health equity. We created a conceptual framework that demonstrates how these factors, supported by community engagement and data, come together to form a business case for mobile health care.

**Discussion:**

Our study demonstrates that mobile clinics can contribute to health care organizations’ business goals by aligning with broader organizational strategies. The conceptual model provides a guide for aligning mobile clinics’ work with business priorities of organizations and funders.

**Conclusions:**

By understanding how health care leaders reconcile the business pressures they face with opportunities to advance health equity using mobile clinics, we can better support the strategic and sustainable expansion of the mobile health sector.

**Supplementary Information:**

The online version contains supplementary material available at 10.1186/s12939-023-01982-8.

## Background

The COVID-19 pandemic has sparked innovation in primary care delivery including expansion of telemedicine, drive-through testing and vaccination sites, and “pop-up” clinics [1–3]. Another innovation that has gained increased attention is the use of mobile health clinics—vehicles customized to facilitate the delivery of care by health care professionals, often in communities underserved by the traditional health care system [4]. While the estimated 2,000 mobile clinics in the United States deliver many types of care, the most common are preventive services and primary care [5]. They are currently funded through a combination of philanthropy, federal and state funding, public and private insurance, and patient payments [5]. Mobile clinics use a variety of strategies to reduce barriers to healthcare, such as employing community health workers and other staff with backgrounds similar to their patients and delivering patient-centered care that emphasized patient education and empowerment [6].

Mobile health programs have been shown to be effective in the management of chronic diseases, such as asthma and hypertension, as well as in the improvement of self-efficacy and participation in recommended screenings [6, 7]. While avoidable emergency room visits and increased use of preventive services reduce health care and societal costs [8], leaders of health care organizations, including public and private payers, clinics, and hospitals, often face more immediate financial pressures [9, 10]. Mobile clinics are funded through a combination of philanthropy, federal and state funding, public and private insurance, and patient payments [5].

Outside the United States, mobile clinics are also used to increase access to healthcare in underserved communities with an emphasis on rural health and responding to humanitarian emergencies [11]. Studies have shown mobile clinics to be effective in diverse settings, including Haiti, Afghanistan, and the Democratic Republic of Congo [12–14].

Our study expands on current understandings of mobile health care as a strategy for improving health outcomes and reducing costs, especially among underserved populations. Understanding how these programs contribute to business-related incentives and disincentives of health care organizations will help providers develop or expand mobile programs to improve population health.

## Methods

We conducted a qualitative thematic analysis to understand the perspectives of 25 health care leaders about business-related incentives and disincentives for mobile health.

Key informants, health care leaders who understand mobile clinics as well as the business pressures facing health care organizations, were identified by an advisory group composed of people with extensive experience in health care delivery. Co-authors (MMW and NEO) developed the interview guide with input from the advisory group.

We used convenience and purposive sampling to recruit key informants by email. The email included information about the purpose of the study and who recommended them as a potential participant. Of the 35 people invited to participate, 25 accepted and were interviewed.

Consent was obtained verbally at the start of the telephone or video conference. Each participant was interviewed one time for approximately 30 min. One author (STB) conducted, recorded, and transcribed all interviews. She had no prior relationship with the participants. Table 1 summarizes participants’ characteristics.

**Table 1:** Interview participants (*N* = 25)

Characteristics	No. (%)
Gender	
Female	15 (60)
Male	10 (40)
Race/Ethnicity	
White	14 (56)
Black/African American	3 (12)
Asian	5 (20)
Hispanic	3 (12)
Geographic Region	
Northeast	16 (64)
Midwest	6 (24)
Southeast	2 (8)
West	1 (4)
Profession/Field	
Physician	8 (32)
Non-profit	13 (52)
Payer	3 (12)
Academia	1 (4)

Beginning with a deductive approach, we used thematic codes derived from a business case framework described by Bailit and Dyer [9]. Three authors (STB, JSL, GHF) independently coded each transcript using MAXQDA v. 20.4.0. During the coding process, additional codes emerged, resulting in a set of deductive and inductive codes developed iteratively through discussions between the coders. Based on these discussions about codes and emerging patterns, the authors determined that saturation had been reached after 23 interviews. Two additional interviews had already been scheduled and were completed for a total of 25.

Using diagramming to reflect on and discuss connections between codes, four authors (STB, JSL, GHF, and MMW) came to a consensus on themes. The coders documented the relationships between codes and themes via detailed notes. Using these diagrams and notes, the researchers iteratively drafted a conceptual framework that visualizes the relationships between the themes. We shared the draft framework with NEO and the advisory group, met to discuss their feedback, made revisions, and shared it with them again before finalizing it.

## Results

We identified 6 themes: (1) organizational culture, (2) business strategy, (3) budget impact, (4) health equity, (5) data, and (6) community engagement.

### Organizational culture

Within the theme, organizational culture, participants described how mobile programs not only contribute to an organization’s mission and communicate that mission to the community, but also reinforce their values within their workforce.

Several participants discussed ways that mobile programs support employee engagement. For example, a business leader for a large health system said the following:

*When we … got approval to do this mobile unit… there was so much excitement around the support that leadership was providing to this new form of care. People were contacting me and knocking on my door to ask how they could be a part of this, which was really exciting. They were so encouraged to see the institution take a new, innovative approach to providing health care.*

A mobile program can bolster efforts to recruit, train, and retain staff by giving trainees, physicians, and other staff opportunities to work more directly with communities. A senior leader within a large network of community health centers shared the following example:

*Experiences that students or other folks have on a mobile unit in a community [are] so valuable in understanding who they’re eventually going to take care of in hospitals.… Without that platform, we wouldn't have recruited those two providers and maybe they would have never kind of realized what their niche is, that community medicine is the thing that they're passionate about.*

### Business strategy

The theme, business strategy, was derived from multiple codes related to healthcare organizations’ business priorities, including new business development and growing the organization’s market share.

Mobile clinics help organizations strategically position themselves in communities as trustworthy and caring. A funder of community health programs, including mobile clinics, described it this way:

*For communities that have been disinvested or marginalized by our health care system through generations. Being able to go to them, make the effort to get right where they are. Say here I am. I'm here to respond to you. It's a good way to be able to bring them into a healthcare system that maybe they are distrustful of.*

For organizations hoping to “expand their reach of getting to new patients, improving compliance, and maybe getting people to switch over to their plans,” mobile clinics offer longer-term business development. One person described the approach used by a colleague’s organization:

*They’ve used their mobile dental clinic to go to a town which doesn’t have a dental clinic, provide services there, build up a patient base, and then apply for a new access point grant through the federal government. They then built a fixed site clinic to create a more permanent resource in that community, then moved their mobile on to another location where they can build up a patient base there as well.*

Other participants described how mobile clinics help with marketing and differentiation in a competitive health care marketplace. They suggested that patients’ experiences with a mobile clinic can influence decisions on where to receive more expensive care: “It cements ‘Whoa wait I need to go to the hospital, I can go into hospital A or I could go to hospital B, but I’m choosing hospital B because I went to their mobile program and they were really nice and took care of me.’”.

### Budget impact

Participants described how mobile clinics can help health care organizations meet their financial goals. Within budget impact, the following subthemes were identified.

#### Start-up costs

Participants explained that some health care decision makers perceive mobile clinics to be more costly due to upfront investment, maintenance, and operational costs, but that actual costs can be lower compared to those for fixed sites. One health care provider argued the following: “From a business standpoint, my starting cost is actually brought down to half, if not more. The ability for us to move from concept to action is much easier.”

#### Adaptability and efficiency

Mobile clinics can adapt to changing needs of populations and are appealing investments for organizations seeking flexibility. One financial leader explained it this way: “When you look at the economics of any particular location — you may have a full-time staff of five, but it's not all five people on all five days. Each town effectively only costs three or four visits a month … so the economics can become pretty attractive.” Mobile clinics can thus be an efficient way to reach patient populations with barriers to health care. He continued: “We can serve those small communities efficiently because we're not there every day of the week. That's where the efficiencies and the economic advantages come up.”

#### Revenue generation

Diverse revenue streams, including insurance, are crucial for mobile clinics. One mobile clinic operator described how specialty care generated revenue for services with lower reimbursement rates, such as primary care:

*We knew that the business case was there, but we were also using cardiology as a way to be able to balance our mission against our margin. ...the point of the mobile is to reach those underserved populations. Although some are not going to make money, we do find ways to be self-sustainable.*

Although many reported insurance reimbursement as an important revenue source, others described difficulties with billing due to state policies or regulations. Some clinics, especially those relying on philanthropy, lacked necessary billing infrastructure: “Billing is a beast. There’s tons of rules. [For a] program that does small volume [it] is really hard.” Others argued that these challenges can be overcome: “We should be charging insurance companies for care that their customers are getting because they’re getting premiums to take care of them.”

#### Value-based care

Mobile programs were viewed as contributors to health quality and value-based care. According to a healthcare provider, mobile health programs can “improve compliance with certain medications and allow for greater follow up and engagement over time,” according to one participant. They added, “A mobile clinic can also help decrease adverse events like heart failure exacerbation, COPD exacerbations, improve A1C scores — things that health systems will be interested in…”.

### Health equity

Often, mobile clinics represent the only accessible and acceptable option for underserved patients. Barriers to care, according to one participant, range from “fear or mistrust of the health care system to immigration status and sometimes just the practicality of getting to a clinic if you have demanding work or family responsibilities.”

Mobile clinics establish patient trust that is difficult to cultivate and worth maintaining once earned. Many interviewees, like the one quoted here, described how mobile clinics build trust with underserved populations:

*For communities that have been disinvested or marginalized by our health care system through generations, being able to go to them, make the effort to get right where they are--say here I am. I'm here to respond to you-- It's a good way to be able to bring them into a healthcare system that maybe they are distrustful of.*

Mobile clinics can reach a broad range of patients, which can reduce gaps in care exacerbated by the COVID-19 pandemic [9]. Many interviewees identify themselves as part of the movement for racial justice. Mobile health programs can help turn intent to address inequities into visible action:

*Communities of color are not getting vaccinated at the same rates and the opportunity to engage and build trust with untrusting communities is huge. It's about not just a connection to a person, but a connection to a community. Certainly, this is also true for homeless communities, for migrant communities, for rural communities...if you can actually come in as an invited guest and a collaborator and work with people, you have just enormous opportunity that you otherwise literally don't have.*

During a precarious time for health care organizations, mobile clinics have demonstrated value not previously recognized. One interviewee noted the following: “Executive leadership in my system this year alone has seen the tremendous unquantifiable value that mobile brings to our community, just by the fact that we have had the capacity and the infrastructure to go out to communities.”

### Data

An additional theme that emerged was related to data. Mobile program leaders often have hunches about financial benefits but need additional data. A participant seeking to expand her mobile program put it this way: “We need more data to look at how many dental related emergencies landed in the emergency room—what was their cost and the cost benefit analysis so we have a data perspective.”

### Community engagement

Participants spoke extensively about community engagement and its importance when planning for new or expanded mobile programs. Participants described the importance of building trust and establishing community partnerships: “People think you get the vehicle and park it and everybody's going to come. That's not always true because they don't know who you are. There was a lot of hesitancy and skepticism as to why we were in their neighborhood.” Interviewees described collaboration with places of worship, libraries, shelters, schools, employers, and other health care organizations as one way to build trust and engage community members.

## Discussion

Business principles increasingly drive health care decisions [15]. As a result, any effort to establish, sustain, or expand a mobile health program must consider the larger business strategy of the parent organization, collaborators, and funders.

While many health care leaders invest in mobile health programs to improve health outcomes, financial stakeholders are required to maintain a business perspective (e.g., lower costs, new revenue) [16]. Mobile clinics treating high volumes of patients with ambulatory care sensitive conditions [17], such as asthma, diabetes, and oral infections, may find it easier to quantify the financial benefit of their efforts to payers or accountable care organizations. However, mobile clinics providing services such as childhood immunizations and cancer screenings—services with longer-term benefits extending beyond the organization that paid for the service and into society and other parts of the health care system—may rely on additional parts of the framework, such as health equity or business strategy.

As the health care landscape continues to change, especially in response to the COVID-19 pandemic, business priorities such as employee engagement and health equity will likely become even more important [18, 19]. Mobile clinics can help health care organizations advance these goals but only if their value is understood by health care leaders.

Funding for mobile healthcare efforts, whether from private or public sources, can be unpredictable, and it can be difficult to sustain a primary care clinic on insurance reimbursements alone, especially if clients are uninsured. In recent years, many local and state governments have declared racism a public health crisis and renewed their commitments to health equity [20]. These declarations must be accompanied by long-term funding commitments and policy changes that value community-based, patient-centered care.

**Fig. 1:**
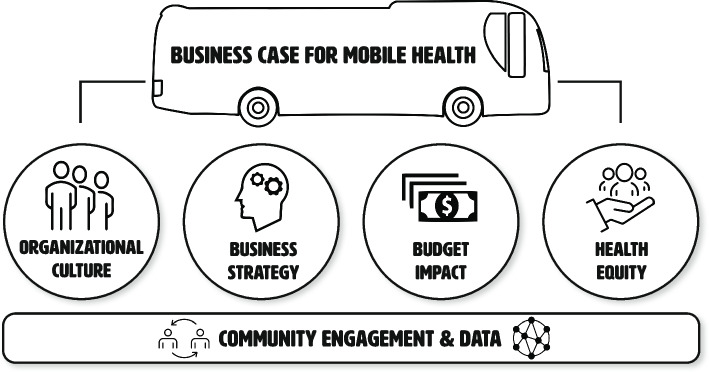
Conceptual framework

The conceptual model (Fig. 1) provides a guide for aligning mobile clinics’ work with business priorities of organizations and funders. There is no single, universal business case for mobile clinics.

Our study has several limitations. Although the qualitative design allowed for exploration of the topic with 25 experts, social desirability is a risk with this methodology. Interviews were conducted during the COVID-19 pandemic and as a result, some key informants were unavailable, and interviewees were likely influenced by the global crisis. We interviewed health care leaders in the United States only and the results may be less relevant in other countries given variations in health care systems, funding mechanisms, and business incentives.

Additional research is needed to substantiate this framework and to further understand each theme. Participants emphasized the importance of research that quantifies budget impact. Case studies exploring how mobile clinics advance business strategies or enhance organizational culture are needed.

## Conclusions

This study elucidates four types of business objectives that mobile health care can support — budget impact, business strategy, organizational culture, and health equity, supported by data and community engagement. By understanding how mobile clinics contribute directly and indirectly to the bottom line of health care organizations, providers and administrators can strategically and sustainably expand the use of mobile programs to improve population health and advance health equity.

### Supplementary Information

**Additional file 1. **

## Data Availability

The datasets generated and analyzed during the current study are not publicly available to protect the confidentiality of participants.
