# Supplementary material for: Health care leaders’ perspectives on the business impact of mobile health clinics
Source: Int J Equity Health. 2023 Sep 1;22:173. doi: 10.1186/s12939-023-01982-8 (PMC10472623; doi:10.1186/s12939-023-01982-8)
Supplement: Supplementary file 1 — Additional file 1. [file 12939_2023_1982_MOESM1_ESM.pdf]

Mobilize Health
Key Informant Interview Guide

Part 1: Background

- Can you tell me a little about yourself and your career?
- What experience do you have with mobile health care?

[IF THE INTERVIEWEE HAS <u>ANY</u> EXPERIENCE WITH MOBILE HEALTH, ASK THE FOLLOWING:]

  - Approximately how long have you worked with mobile health programs?
  - In your current role, are you responsible for any aspect of mobile health care? If so, what is your role?
  - What types of services does your clinic offer? (e.g. mobile mammography, primary care, oral health)

Part 2: General Benefits and Disadvantages of Mobile Health

- In your opinion, what are the benefits of mobile health care?
- What do you see as the disadvantages or obstacles to mobile health care?

Part 3: Financial Incentives and Disincentives

We are exploring the business side of mobile health care, the incentives and disincentives experienced by health systems, payors, and government agencies when starting or expanding mobile health programs.

- Let's start with the incentives. In your opinion, what are some of the financial benefits or incentives to provide mobile health care?
  - PROMPTS IF THE INTERVIEWEE DOES NOT READILY IDENTIFY INCENTIVES:
    - Have you seen changes in incentives as we move away from a fee-for-service system to one based on performance and patient outcomes?
    - Have you considered improvements in appropriate use of health care resources, such as reducing avoidable visits to the emergency department?
    - What about incentives that have an indirect impact on the bottom line, such as public relations, patient engagement, or increased market share?
- What about disincentives? From a financial perspective, why might an organization decide <u>not</u> to start or expand a mobile health program?

- Has the COVID-19 pandemic changed any of these incentives or disincentives? If yes, how?

Part 4: Current Financial Situation [ASK ONLY OF PEOPLE WHO ARE CURRENTLY INVOLVED WITH MOBILE HEALTH CARE DELIVERY]

- Can you tell me about your organization?

  [IF NOT ALREADY ANSWERED, ASK THE FOLLOWING:]
  - For-profit or non-profit?
  - Affiliated with hospital or community health center? Independent?
  - [If affiliated with a hospital system, ask:] Where within the health care system's organization, does the mobile clinic reside? (e.g. community benefit, population health management, radiology, foundation, etc)
  - Do you bill insurance for services delivered by the mobile clinic?
  - What other funding sources do you rely on (e.g. philanthropy, corporate sponsorship, government grants, etc.)

- Does your clinic normally generate a profit, does it run a deficit, or break even?

- To what do you attribute these financial results?

Part 5: Summary Questions

- I have two final questions. Imagine you are trying to persuade a health care leader, such as the CEO of a hospital or a state Medicaid director, that mobile health care makes financial sense. What would you say to them?

- Now I want you to take the opposite stance. Imagine a health care leader is considering starting or expanding a mobile health program. What would you say to persuade them to not move forward?

- Is there anything else you think we should know or consider?
